# Trends in output of hypertension management and associated factors in primary care facilities: a latent trajectory analysis in China from 2009 to 2017

**DOI:** 10.1186/s12875-023-02139-w

**Published:** 2023-09-06

**Authors:** Lei Duan, Liang Zhang, Xiang Zhang, Shan Lu

**Affiliations:** 1 School of Medicine and Health Management, Tongji Medical College, Huazhong University of Science and Technology, Wuhan, 430030 China; 2 Research Centre for Rural Health Service, Key Research Institute of Humanities & Social Sciences of Hubei Provincial Department of Education, Wuhan, 430030 China; 3 School of Political Science and Public Administration, Wuhan University, Wuhan, 430072 China

**Keywords:** Hypertension management, Output, Primary care, Latent class growth analysis, China

## Abstract

**Background:**

The prevalence of hypertension is high (25.2% in 2012) and there were a large number of patients with hypertension (more than 200 million) in China. Township health centres in rural areas and community health centres in urban areas are responsible for hypertension management. This study aims to identify trends in hypertension management output and related facility-level, geographical and economic factors in primary care facilities and to assess the effect of the national project of basic public health services in China from 2009 to 2017.

**Methods:**

A cross-sectional survey (2018) was combined with retrospective data collection (2009–2017) from 685 primary care facilities in six provinces in China. The hypertension management output was indicated by the number of patients with hypertension under management per 10,000 population. Latent class growth analysis and group-based trajectory models were applied to classify trajectories and determine associations with facility-level, geographic and economic characteristics.

**Results:**

The trend in the output increased rapidly from 2009 to 2012 with an average growth rate of 54.58% and slowed down from 2012 to 2017 (growth rate of 5.94%). Five trajectories of the output were identified and labelled according to baseline status and increase rates: low-gradually increasing (16.9%), middle-slightly increasing (16.2%), low-sharply increasing (7.9%), middle-sharply increasing (34.2%) and persistently high (24.9%). The time-stable characteristics, including region (eastern, central or western), district (urban or rural), landform, were associated with hypertension management output of the facilities. Number of public health physicians was a significant time-dependent characteristic influencing management output.

**Conclusions:**

Five latent trajectories of hypertension management output were identified. The output was still at a low level compared with the prevalence of hypertension. Hypertension screening in young people need to be emphasized. Facilities are recommended to establish good relationships with residents for better hypertension management outcomes especially in urban areas.

## Introduction

Hypertension is a major modifiable risk factor for stroke and other cardiovascular diseases [1]. Blood pressure levels are increasing driven by population aging, urbanisation and rising incomes in China, which can be largely attributed to the high stroke mortality [2, 3]. According to the Seventh National Population Census: 18.70% of the population is over 60 years old and 13.50% is over 65 years old in China in 2020. Surveys indicated that hypertension is highly prevalent, which was 25.2% in 2012 according to the China Health Statistics Yearbook, but the awareness, treatment and control rates of hypertension remain low in China across studies [1, 3–7].

China launched a new round of health care reform in 2009, giving priority to public health strengthening [8]. Chronic disease management, including hypertension and diabetes management, was then added to the package of basic public health services. Blood pressure control, which used to be the aim of local research projects organised by experts, became a national public health priority led by the government [9]. The service scope of disease management for patients with hypertension is designed by the government, including (i) screening, (ii) follow-up, (iii) classified intervention/treatment and (iv) medical examination. All basic public health services are provided to all residents for free at primary care facilities which play an important role in the prevention and treatment of hypertension [10, 11]. Primary care facilities can assist patients with hypertension in leading healthier lives, thereby reducing the burden of hypertension-related complications and improving the efficiency of medical resource utilization in healthcare systems. Previous studies showed that patients registered and managed by primary care facilities would have better control of blood pressure and significant decrease of related complication [12].

Though previous surveys (conducted after the health care reform) revealed that China achieved improvements in awareness, treatment and control rates of hypertension, which were nearly two, two and three times as that in 2002, respectively, [4, 13, 14] the rates were still low compared with high-income countries [4]. Moreover, there were geographic variations in hypertension management with regard to the management rate, quality of services and patient or provider satisfaction [15–17]. Most studies used cross-sectional data to present the status of the hypertension epidemic and compared the results with previous surveys. Research on trajectories of hypertension management using longitudinal data is lacking, which may be helpful to understand and predict dynamic trends. Previous studies analysed variations at the regional level (e.g. Eastern/Central/Western China) or provincial level, but to our knowledge, no study has examined the variations at the facility level. Results have proved that primary care facility characteristic (e.g. having more primary care physicians with at least a bachelor’s degree) is associated with hypertension awareness, treatment and control rate [3].

China implemented the national basic public health services project to introduce hypertension management services in 2009, so an understanding of the output of hypertension management which is an important indicator for project assessment and will be the basis of quality of care in primary care facilities is important. The National Basic Public Health Service Project mandates that primary care facilities should carry out free annual blood pressure screening for residents aged 35 and above living in the jurisdiction and provide health management and physical examinations for patients with hypertension. This study evaluated the output of hypertension management in basic public health services projects between 2009 and 2017, and explore the associated factors at primary care facilities to provide a basis for improving output of hypertension management.

We address the following research questions specifically:

1. How has the output of hypertension management developed in China’s basic public health services projects from 2009 to 2017?
2. What are the latent class characteristics of output of hypertension management at primary care facilities between 2009 and 2017?
3. What are the economic, geographical and facility-level characteristics associated with the output of hypertension management at primary care facilities?

## Methods

### Study design and sample

**Fig. 1:**
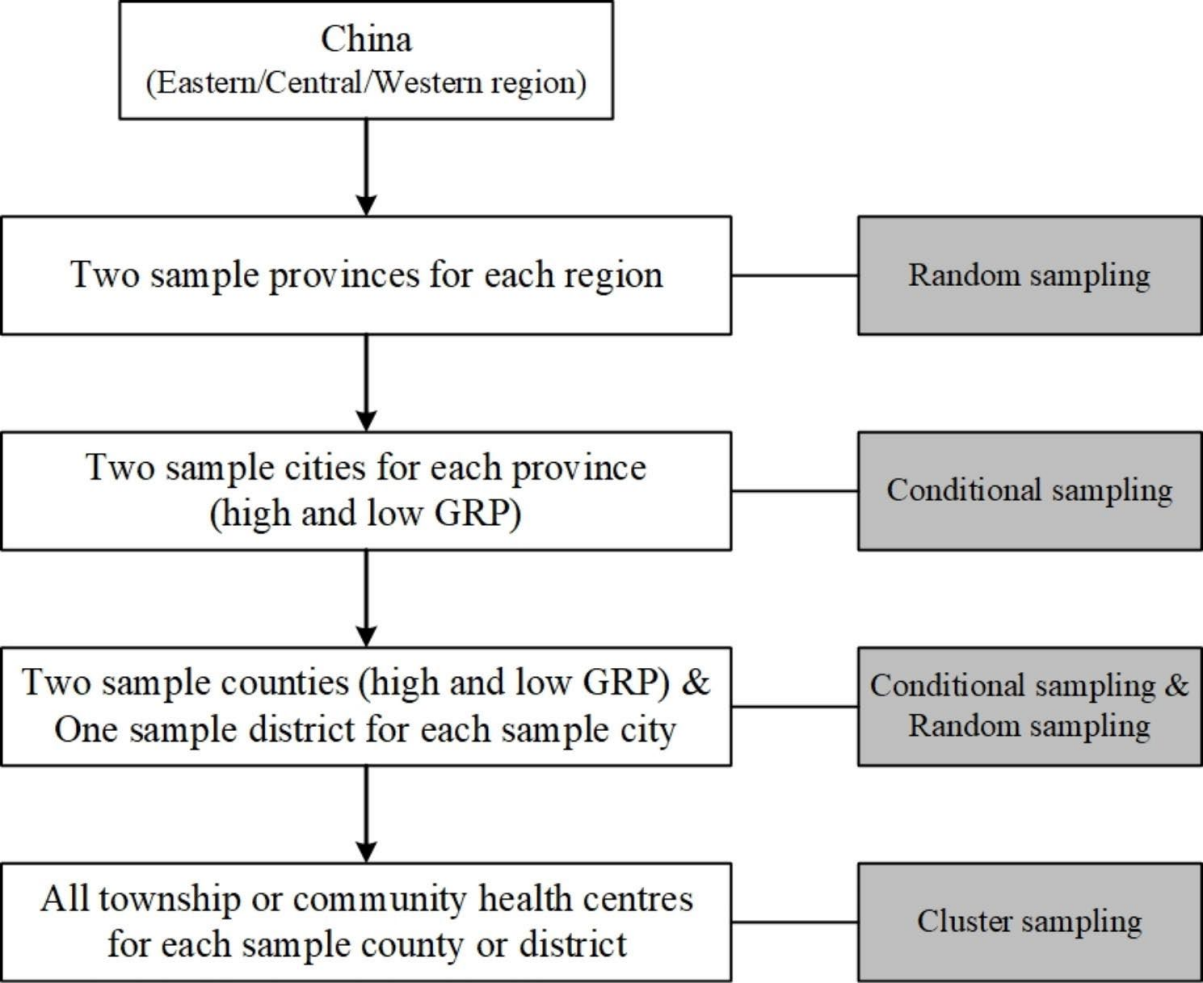
The sampling flowchart and methods

A cross-sectional survey (2018) amongst primary care facilities was combined with retrospective data collection from the Health Statistical Annual Reports (2009–2017) for each participating primary care facility. Multistage stratified cluster sampling was used to determine the samples of primary care facilities from eastern, central and western China. The sampling flowchart and sampling methods are shown in Fig. 1. Two sample provinces in each region were selected, and two cities in each province were selected according to gross regional product (GRP) per capita (high and low). Given that the number of counties (representing rural areas) was twice that of districts (representing urban areas) in China, two sample counties (with a relatively high and low GRP per capita) and one sample district were randomly selected from each sample city [8]. We identified 24 counties and 12 districts in summary; all township and community health centres in the selected sample counties and districts were included for investigation. The sample of primary care facilities is presented by Table 1. In total, 720 primary care facilities were surveyed and their Health Statistical Annual Reports were collected, and 685 primary care facilities were left after cleaning the abnormal values or missing data with an effective response rate of 95.14%, which included 416 township health centres and 269 community health centres. We excluded the sample if it had missing data for years in the survey database and Health Statistical Annual Reports System. As it is used to fit the trend of the change, the program allows for one or two years of missing values in an institution’s data. There should be 200 samples at least to ensure the identification rate according to previous studies [18, 19].

**Table 1:** Study sample of primary care facilities

Region	Sample provinces	Sample cities	Districts and counties
Eastern China (12 provinces and municipalities)	Guangdong	Shenzhen	2 districts ^1^
		Shaoguan	4 countries ^1^
	Shandong	Qingdao	1 district and 2 counties
		Jining	1 district and 2 counties
Central China (9 provinces)	Hubei	Yichang	1 district and 2 counties
		Huanggang	1 district ^2^ and 2 counties
	Henan	Luoyang	1 district and 2 counties
		Shangqiu	1 district ^2^ and 2 counties ^2^
Western China (10 provinces and municipalities)	Guizhou	Zunyi	1 district and 2 counties
		Tongren	1 district and 2 counties
	Chongqing ^3^	Chongqing	2 districts and 4 counties
Count	6 provinces and municipality	11 cities	12 districts and 24 counties

^1^ Shenzhen city has no counties, and thus we selected 2 districts from Shenzhen city and 4 counties from Shaoguan city for Guangdong province. ^2^ The Health Statistical Annual Reports (2009–2017) for participating primary care facilities from a district in Huanggang city and one district and one county in Shangqiu city were missing. ^3^ Chongqing is a municipality directly under the central government in China and a provincial-level city in China. We selected 2 districts (with relatively high GRP per capita) and 4 counties (2 with relatively high GRP per capita and 2 with low GRP per capita) in total

### Measures

#### Output of hypertension management

The output of hypertension management was defined by the number of patients with hypertension, who received hypertension management according to the ‘National Standards for Basic Public Health Services’ at the primary health facilities in eastern, central, and western China per 10,000 population. The standards contain a form of follow-up service record for patients with hypertension, which provide the data records and evidence for counting. The number of patients with hypertension who were under management during this year and the number of resident population under service were derived from the Health Statistical Annual Reports from 2009 to 2017, which were downloaded from the National Health Statistics Reporting System. Through this system, each primary care facility is required to submit a yearly report to record its resources and volume of services at the end of each year, so we were able to calculate the output of hypertension management accurately.

#### Associated factors

The associated factors included in this study can be classified into three categories, namely, facility-level, economic and geographic characteristics. Firstly, facility-level characteristics included the number of public health providers and the number of primary care providers per 10,000 population, which were used to represent the public health workforce, and obtained from the National Health Statistics Reporting System. The public health providers are full-time public health physicians working at primary care facilities, and primary care providers refer to all healthcare technical personnel including physicians, nurses, pharmacists, clinical laboratory technicians and imaging technicians working at primary care facilities. Secondly, economic characteristics were measured by GRP per capita of a county or district, which was calculated by the data from regional economic and social yearbooks of 36 counties or districts for 9 years. Thirdly, geographic characteristics included region (eastern, central or western China), rural or urban, landform (plain, plain and hilly, hilly and mountainous and mountainous) and service radius (distance in kilometre from the primary care facility to the farthest family within the catchment area), which were obtained from the cross-sectional survey. All facility characteristics were standardised by their standard deviation.

### Statistical analysis

A descriptive figure on changes in output of hypertension management was provided. Latent class growth analysis (LCGA) was conducted to classify the trajectories of the output of hypertension management into different groups [20]. The distribution of hypertension management output was slightly skewed, so log-transformed data were used as dependent variables for LCGA. We examined the association between groups and various time-stable (i.e. geographic) or time-dependent (i.e. facility-level and economic) characteristics. To determine the number of trajectory categories, the Bayesian information criteria (BIC) and the proportion of each trajectory categories were considered [20]. LCGA and analysis of time-dependent characteristics were performed using the PROC TRAJ procedure (Jones & Nagin, Pittsburgh, PA, USA), and analyses of time-stable characteristics for hypertension management output were carried out using Chi-square test and ANOVA test. All analyses were conducted in Stata 15.1. Statistical significance was set to two-tailed p < 0.05.

### Patient and public involvement

This study does not involve patients or public. The research unit of this study is primary care facility.

## Results

### Changes in output of hypertension management from 2009 to 2017

**Fig. 2:**
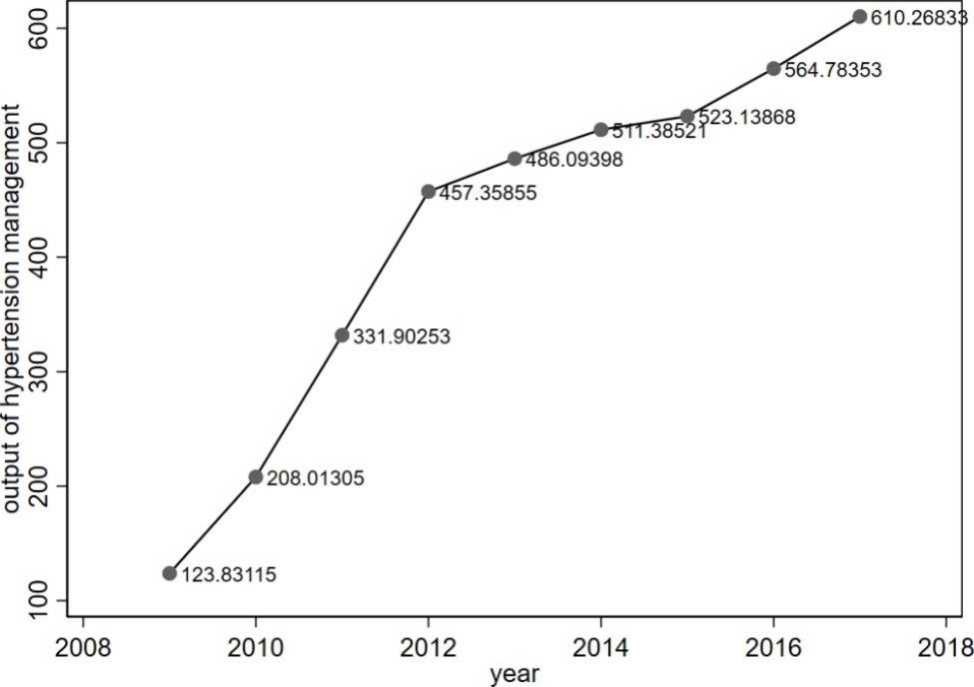
Changes in average output of hypertension management amongst primary care facilities from 2009 to 2017

Figure 2 shows the trend in average output of hypertension management from 2009 to 2017. The average output for all of the facilities each year increased from 123.83 to 610.27 per 10,000 population (with a growth rate of 492.83%). It increased rapidly from 2009 to 2012 and slowed down from 2012 to 2017. The average growth rate of output was 54.58% from 2009 to 2012 and 5.94% from 2012 to 2017.

### Trajectories of the output of hypertension management

We examined 2- to 6-class models. BICs indicated that the model fit better with a higher number of classes. However, the 6-class model included a class that only accounted for 2.6% of the samples, which was relatively small to be accepted, and trajectories 1 and 2 in the 6-class model shared the same start point and finish level. Therefore, we selected the 5-class model that identified five trajectories as the best fit on the basis of model fit statistics and proportion of each class.

**Fig. 3:**
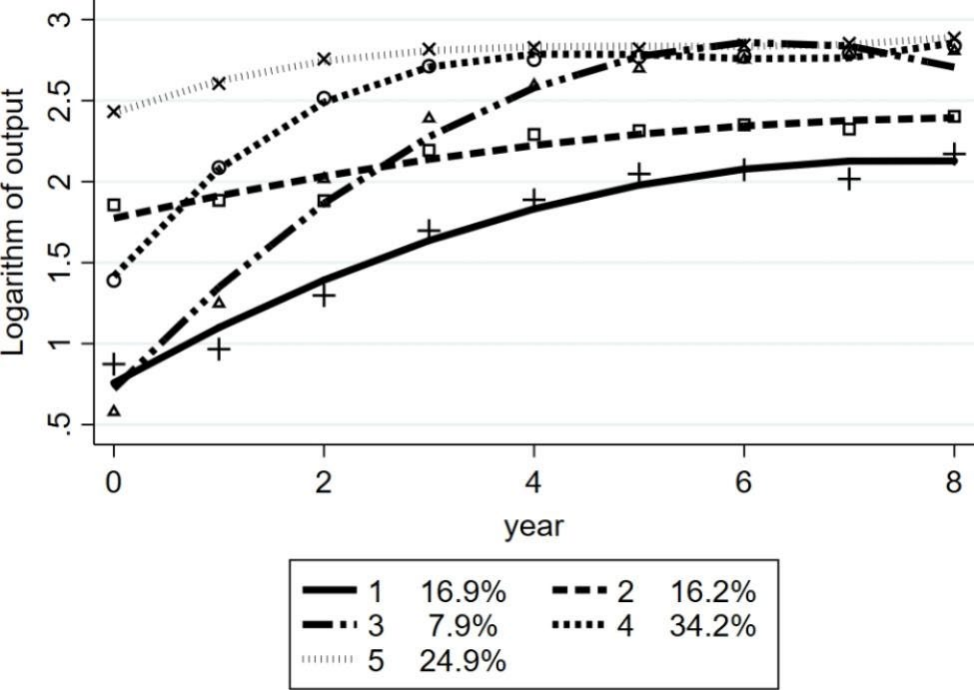
Trajectories of the log-transform output for hypertension management rate (*n* = 685)

Figure 3 illustrates the trajectories of the logarithmic output for hypertension management at primary care facilities. We used the start point and trend to describe the trajectory as follows. Trajectories 1 (16.9%) and 3 (7.9%) were ‘low-gradually increasing’ and ‘low-sharply increasing’, respectively, sharing the same start point but differing in finish levels. Trajectory 2 (16.2%) was ‘middle-slightly increasing’ whilst being persistently middle for 9 years. Trajectory 4 (34.2%) was ‘middle-sharply increasing’, which showed that the output sharply increased from a median to a high level. Trajectory 5 (24.9%) was ‘persistently high’, showing that the output was relatively stable and only slightly increased from 2009 to 2013.

### Distribution in trajectories with time-stable characteristics

Table 2 presents the distribution of the primary care facilities with different time-stable characteristics. Facilities in eastern and urban areas had a higher percentage in trajectories 1 and 2. About 80% of the facilities in trajectories 3, 4 and 5 were located in rural areas. Besides, the majority of the facilities in trajectories 1 and 2 were located in plain areas and had a short service radius. Table 2 also shows that facilities within different trajectories had significant statistical difference with the time-stable characteristics (*p* < 0.001).

**Table 2:** Distribution of the facilities with different time-stable characteristics in five trajectories

Trajectory	1 (n = 117)	2 (n = 108)	3 (n = 48)	4 (n = 260)	5 (n = 152)	Count (n = 685)	Statistical values	p value
Region:								
Eastern	103 (88.03%)	81 (75.00%)	11 (22.92%)	83 (31.92%)	47 (30.92%)	325 (47.45%)	186.103υ	< 0.001*
Central	1 (0.85%)	5 (4.63%)	6 (12.50%)	53 (20.38%)	49 (32.24%)	114 (16.64%)		
Western	13 (11.11%)	22 (20.37%)	31 (64.58%)	124 (47.69%)	56 (36.84%)	246 (35.91%)		
District:								
Urban	104 (88.89%)	81 (75.00%)	5 (10.42%)	53 (20.38%)	26 (17.11%)	269 (39.27%)	265.549υ	< 0.001*
Rural	13 (11.11%)	27 (25.00%)	43 (89.58%)	207 (79.62%)	126 (82.89%)	416 (60.73%)		
Landform:								
Plain	73 (62.39%)	80 (74.07%)	9 (18.75%)	68 (26.15%)	57 (37.50%)	287 (41.90%)	131.915υ	< 0.001*
Plain & hilly	21 (17.95%)	4 (3.70%)	3 (6.25%)	31 (11.92%)	21 (13.82%)	80 (11.68%)		
Hilly & mountainous	12 (10.26%)	7 (6.48%)	23 (47.92%)	87 (33.46%)	44 (28.95%)	173 (25.25%)		
Mountainous	11 (9.40%)	17 (15.74%)	13 (27.08%)	74 (28.46%)	30 (19.74%)	145 (21.17%)		
Service radius (km):								
Mean (SD)	2.39 (4.60)	5.25 (16.08)	9.55 (6.39)	17.97 (24.79)	18.39 (34.46)	12.81 (24.23)	14.238υυ	< 0.001**
GRP per capita (1000 CNY)***:								
Mean (SD)	108.45 (42.73)	155.40 (93.25)	40.84 (16.28)	55.24 (40.75)	61.66 (36.76)	80.54 (63.88)	67.643υυ	< 0.001**

*p value of Chi-square test to examine the significance in the characteristics amongst the trajectories

** p value of ANOVA test to examine the significance in the characteristics amongst the trajectories

*** GRP in 2017 was used for calculation and analysis

υ Chi-Square values of the chi-square test

υυ F-value of the ANOVA

### Facility-level, economic and geographic characteristics associated with the changes

Table 3 provides time-stable characteristics associated with the trajectories of the hypertension management output. Primary care facilities in central and western areas had higher probabilities to fall under trajectories 3, 4 or 5 compared with trajectory 1. This trend also applied to rural areas and large service radius.

**Table 3:** Time-stable characteristics associated with different trajectories of hypertension management output

Characteristics	β	SE	p value
1.Trajectory 1 (low-gradually increasing) (reference)			
2.Trajectory 2 (middle-slightly increasing)			
Constant	-0.095	0.197	0.628
Region: Eastern (reference)			
Central	1.511	1.263	0.231
Western	2.261	0.792	0.004
District: Urban (reference)			
Rural	0.707	0.668	0.290
Landform: Plain (reference)			
Plain and hilly	-2.801	0.808	< 0.001
Hilly and mountainous	-2.497	0.872	0.004
Mountainous	-1.493	0.846	0.078
Service radius	3.036	1.656	0.067
3.Trajectory 3 (low-sharply increasing)			
Constant	-3.199	0.587	< 0.001
Region: Eastern (reference)			
Central	3.302	1.268	0.009
Western	3.105	0.866	< 0.001
District: Urban (reference)			
Rural	2.997	0.838	< 0.001
Landform: Plain (reference)			
Plain and hilly	-1.101	0.927	0.235
Hilly and mountainous	-0.281	0.839	0.738
Mountainous	-1.463	0.999	0.143
Service radius	3.575	1.641	0.029
4.Trajectory 4 (middle-sharply increasing)			
Constant	-2.118	0.399	< 0.001
Region: Eastern (reference)			
Central	3.173	1.123	0.005
Western	2.433	0.702	< 0.001
District: Urban (reference)			
Rural	2.525	0.653	< 0.001
Landform: Plain (reference)			
Plain and hilly	-0.018	0.550	0.974
Hilly and mountainous	-0.119	0.669	0.859
Mountainous	-1.190	0.790	0.132
Service radius	5.128	1.571	0.001
5.Trajectory 5 (persistently high)			
Constant	-1.671	0.300	< 0.001
Region: Eastern (reference)			
Central	4.329	1.100	< 0.001
Western	2.665	0.726	< 0.001
District: Urban (reference)			
Rural	2.315	0.659	< 0.001
Landform: Plain (reference)			
Plain and hilly	-1.069	0.560	0.056
Hilly and mountainous	-1.410	0.682	0.039
Mountainous	-2.473	0.809	0.002
Service radius	5.132	1.572	0.001

Table 4 provides time-dependent characteristics associated with the trajectories of the hypertension management output. The number of public health physicians per 10,000 population was associated with an increase in all the groups, except the middle-sharply increasing group (β 0.008, *p* = 0.554). The number of primary care providers was associated with an increase in output in the middle-slightly increasing (β 0.106, *p* < 0.001), low-sharply increasing (β 0.106, *p* < 0.001) and persistently high groups (β 0.083, *p* < 0.001) but a decrease in the middle-sharply increasing groups (β -0.048, *p* < 0.001). No association was observed in the low-gradually increasing group (β 0.071, *p* = 0.254). The economic characteristic GRP per capita was associated with an increase in output in the middle-sharply increasing group (β 0.122, *p* < 0.001) but showed a decrease in the other groups.

**Table 4:** Time-dependent characteristics associated with different trajectories of hypertension management output

Characteristics	β	SE	p value
1.Trajectory 1 (low-gradually increasing)			
Primary care providers	0.071	0.062	0.254
Public health provider	0.225	0.038	< 0.001
GRP per capita	-0.368	0.035	< 0.001
2.Trajectory 2 (middle-slightly increasing)			
Primary care providers	0.106	0.014	< 0.001
Public health provider	0.131	0.016	< 0.001
GRP per capita	-0.417	0.023	< 0.001
3.Trajectory 3 (low-sharply increasing)			
Primary care providers	0.106	0.012	< 0.001
Public health provider	0.048	0.007	< 0.001
GRP per capita	-0.066	0.014	< 0.001
4.Trajectory 4 (middle-sharply increasing)			
Primary care providers	-0.048	0.011	< 0.001
Public health provider	0.008	0.013	0.554
GRP per capita	0.122	0.021	< 0.001
5.Trajectory 5 (persistently high)			
Primary care providers	0.083	0.009	< 0.001
Public health provider	0.026	0.007	< 0.001
GRP per capita	-0.112	0.008	< 0.001

## Discussion

This study reveals that there was a rapid increase in the hypertension management rate from 2009 to 2012, but the growth slowed down from 2012 to 2017. We identified five distinct trajectories of hypertension management output in primary healthcare facilities from 2009 to 2017 in China and labelled them based on start point and increase rate. Facilities with trajectories of low-sharp increase, middle-sharp increase, and persistently high were more commonly found in central and western rural areas of China. Additionally, the study found that geographic characteristics, including region, urban-rural classification, and landform, as well as facilities-level and economic characteristics, were significantly associated with different trajectories.

The implementation of the national project of basic public health services has resulted in a continuous increase in hypertension management output. Figure 2 shows that the growth rate of output exhibited a turning point in 2012. As the project was just beginning to be implemented in 2009, a large number of patients with hypertension availed of health management services during the first few years, which led to a rapid increase in output. Growth slowed down from 2012 to 2017, which indicated the saturation point in standardised management of the existing patients with hypertension. However, previous survey data showed a large gap between the current number of people under management and the total number of patients with hypertension. According to the China Health Statistics Yearbook, there were 2520 patients with hypertension in a population of 10,000 [21]. This study showed that only 610.27 patients with hypertension were managed per 10,000 population in 2017, which was the maximum output. The possible reason for such a large gap lies in the low awareness rate of patients to their own hypertension disease [16, 22, 23]. A previous study showed that the crude hypertension awareness rate was 43.8% and the age-standardised hypertension awareness rate was only 27.2% in 2015 [24]. As we cannot obtain the data of the hypertension awareness rate of the same population from the sample areas, we used the data from a nationally representative survey to validate our reasoning. It revealed that a significant number of patients with hypertension had not undergone screening and detection, or if they had, they had not received adequate health management. The survey data also revealed that the output of hypertension management was stable after 2012. The average growth rate of hypertension management output was 5.94% from 2012 to 2017, which was slightly lower than the rate of increase in prevalence. A study from China Health and Nutrition Survey showed that the average increase rate of prevalence of hypertension was 7.73% during 2011–2015 [25]. This result indicated that patients with new-onset hypertension had not been managed completely. The gap may continue to grow between the number of patients with hypertension under management and the total patients with hypertension. Therefore, we suggest that primary healthcare facilities implement health education programs, disease screening, and other measures to improve the awareness and health literacy of patients with hypertension. Additionally, we recommend optimizing the allocation of human resources in primary care facilities and improving the accessibility of health services to promote the management of patients with new-onset hypertension.

On the basis of the trajectories of the output in Fig. 3, low-sharply increasing, middle-sharply increasing and persistently high groups, which accounted for over 60% of the total facilities, presented high-level output in the last 4 years of the studied time period. Table 2 revealed that facilities in rural areas had a higher percentage in these trajectories, which indicated that the majority of rural area facilities demonstrated good performance in the output. The result of good performance in the output in central and western rural areas might be related to the relationships and economic factors. Residents in rural areas had a closer relationship with village doctors, who were the actual operators of health management on behalf of township health centres in China. Research shows that social relationships could contribute to higher hypertension management outcomes, [26] just like the social network formed between village doctors and residents in rural China. Urban and eastern rural residents were relatively better off so that they usually choose high-level healthcare facilities when needed, and thus the primary healthcare facilities did not have a comprehensive picture of the health status of residents in jurisdictions to decide who needs to be included in health management. According to former studies, the prevalence of management and control of hypertension in urban areas were higher than that in rural areas in China [7, 14, 27]. As a result, the higher output in rural areas did not mean higher treatment rates or control rates of hypertension, the reason for the gap might be the quality of the management; [28–31] facilities in rural areas managed patients with hypertension simply by filing cards sometimes without follow-up visits [31, 32]. Therefore, we recommend that township hospitals improve management quality to increase the prevalence of control and decrease or delay complications by increasing the frequency of follow-up visits and medication guides [33]. By comparison, facilities in urban areas had lower management output, which might be related to the self-medication [34] and the disconnect of the information system between hospitals and primary care facilities [35]. Urban residents sought treatment freely because of more hospitals in urban areas, and the lack of trust in primary care facilities led to bypassing community health centres [36]. The reactive service-providing model and information between hospitals and primary care facilities was not exchanged demonstrated that primary care facilities could not fully grasp the disease situation of patients with hypertension in their catchment area [31]. Therefore, we recommend that primary care facilities in urban areas carry out additional hypertension screening, and the information between hospitals and primary care facilities should be interconnected.

Trajectory trends showed that facilities in the low-gradually increasing and low-sharply increasing groups shared the same start point but differed in growth rate. Facilities in the low-sharply increasing groups were more likely to be located in central, western and rural areas [37]. With the rapid economic and social development, a demographic change occurred in both urban and rural areas in China [1]. The process of urbanisation promoted the migration of young population in rural areas to urban areas, and the proportion of the elderly population in rural areas kept rising, as well as the proportion of patients with hypertension [38, 39]. As a result, the output of hypertension management of primary care facilities in rural areas increased rapidly. We recommend that facilities in rural areas should increase resource input in public health services to adapt to the rapidly increasing demand for chronic disease management. According to the existing studies, limited health personnel is one of the health system-related barriers to the appropriate control of hypertension, [40] but the trend of its impact over time has not been discovered. The role of human resources was critical in hypertension management in primary care facilities, even for non-physician healthcare service providers [41]. Healthcare service providers in primary care facilities were divided into public health service providers and other providers in China. Although the number of public health service providers contributed to the output, the current growth in the number of public health service providers cannot keep pace with the growth in services; the proportion of public health service providers is much lower than that of clinical service providers in facilities, whereas the proportion of public health services is much higher than that of medical services [8]. Primary care facilities paid more attention to medical services than public health services, resulting in the decline in quality of public health services [42]. We recommend increasing the input on human resources in grassroots facilities and maintaining and gradually improving the number of full-time public health personnel. It showed that the coefficient of the low-sharply increasing group (0.048) was lower than that of the low-gradually increasing group (0.225), and Wald test [20] showed a significant statistical difference between the two groups (P < 0.0001). Therefore, facilities in the low-sharply increasing group completed more hypertension management tasks with fewer public health service providers and higher growth rates, which resulted in heavy workload of public health providers and poor performance [43]. However, most of these facilities were concentrated in rural areas, which indicated some problems in management quality as patients with hypertension were not managed comprehensively and to a high standard [31]. The effective development of public health services in primary care facilities should be encouraged through policy and special subsidies for public health services [44, 45] to improve the output and standardisation of hypertension management.

Given the limitation in accessing the number of patients with hypertension, we employed management output instead of management rates to assess performance. Thus, we normalised the volume of services using the population of the area as the output, assuming that different regions shared the same prevalence of hypertension. We also determined the trend of management output to describe changes. We discovered that the increasing trend of hypertension management output was valuable to assess the effect of the national project as the project was implemented in 2009 and hypertension management was still at a low level.

Strengths of this study include that large sample and longitudinal data of primary care facilities for 9 years in China were involved and longitudinal data analysis methods were used to analyse the output of hypertension management. We analysed both time-dependent and time-stable characteristics influencing the trend of the hypertension management output. Given the limitation in accessing the number of patients with hypertension, we employed management output instead of management rates to assess performance.

## Conclusion

Since the implementation of national basic public health service project in 2009, hypertension management output increased rapidly in China. However, the output of hypertension management was still at a low level, indicated that not all of the patients with hypertension were managed, and the gap between the number of patients managed and the total number of patients might become increasingly larger. Currently, hypertension management services are only provided to key populations in China, indicating that there is still a significant need to improve the output and accessibility of hypertension management services. We recommend to expand the population coverage of free national physical check-up and hypertension screening to improve the awareness rate of patients with hypertension and help the facilities to grasp the health status of residents in the jurisdiction. Besides, the quantity and quality of public health service providers need to be improved.

## Data Availability

The datasets generated and/or analysed during the current study are available from the investigation of the research funded by the National Natural Science Foundation of China (Grant no: 71,734,003) but restrictions apply to the availability of these data, which were used under license for the current study, and so are not publicly available. Data are however available from corresponding author on reasonable request and with permission of the project principal.
